# Evaluation of PCR based DNA sequencing targeting mitochondrial larger subunit (mtLSU) region for the rapid detection of *Pneumocystis jirovecii* in sputum from HIV positive patients

**DOI:** 10.1186/1471-2334-12-S1-P78

**Published:** 2012-05-04

**Authors:** M Revathy, K Lily Therese, C Chandrasekar, J Suria Kumar, HN Madhavan, A Kamala Vinayagam

**Affiliations:** 1 L & T Microbiology Research Center, Vision Research Foundation, Sankara Nethralaya Chennai, India; 2 Government Hospital of Thoracic Medicine, Chennai, India

## Background

To establish rapid and reliable diagnostic techniques for the detection and identification of *Pneumocystis jirovecii* (*P. jirovecii*) as there are no standard laboratory diagnostic techniques available for *Pneumocystis jirovecii* Pneumonia (PJP) infections.

## Materials and methods

A total of 104 sputum specimens from HIV positive and clinically suspected PJP patients attending Government Hospital of Thoracic Medicine during Dec 2009-Mar 2011 were included in the study. Direct staining of sputum specimens using KOH Calcoflour and Grocott methanamine silver staining (GMS) and nested PCR targeting mitochondrial larger subunit region (mtLSU) was optimized. The optimized PCR was applied on all clinical specimens and amplified products were subjected to DNA sequencing and the sequencing results were analyzed using BLAST analysis software to deduce the sequence homology.

## Results

Of the 104 clinical specimens included in the study, *P. jirovecii* was detected in 35 sputum specimens (36.4%) by KOH Calcoflour and GMS staining. Nested PCR using primers targeting the mtLSU region was specific and sensitive to detect 500fg of *P. jirovecii* DNA with base pair product size of 346 for first round and 120bp for the second round. The optimized PCR was applied on all sputum specimens, resulting in 47.84% (46/104) positivity for detecting the genome of *P. jirovecii*. The DNA sequencing of the amplified PCR products showed 98-100% sequence similarity with *P. jirovecii* deposited in the genbank.

## Conclusions

Nested PCR based DNA sequencing targeting mtLSU region is a sensitive, specific, rapid and reliable laboratory diagnostic tool for the detection of PJP directly from sputum specimens.

